# RNA-seq analysis to identify genes related to resting egg production of panarctic *Daphnia pulex*

**DOI:** 10.1186/s12864-023-09369-3

**Published:** 2023-05-17

**Authors:** Natsumi Maruoka, Takashi Makino, Jotaro Urabe

**Affiliations:** 1 Graduate School of Life sciences, Tohoku University, 6-3 Aoba, Aramaki, Aoba-ku, Sendai, 980-8578, 022-795-6686 Miyagi Japan; 2 Center for Bioscience Research and Education, Utsunomiya University, 350 Mine-machi, Utsunomiya, 321-8505, 028-649-5129 Tochigi Japan

**Keywords:** Diapause, RNA-seq, *Daphnia*, Resting egg, Obligate parthenogenesis

## Abstract

**Background:**

The genus *Daphnia* switches its reproductive mode from subitaneous egg production to resting egg production in response to environmental stimuli. Although this life history trait is essential for surviving unsuitable environments, the molecular mechanism of resting egg production is little understood. In this study, we examined genes related to induction of resting egg production using two genotypes of panarctic *Daphnia pulex*, the JPN1 and JPN2 lineages, which differ genetically in the frequency of resting egg production. We reared these genotypes under high and low food levels. At the high food level, individuals of both genotypes continually produced subitaneous eggs, whereas at the low food level, only the JPN2 genotype produced resting eggs. Then, we performed RNA-seq analysis on specimens of three instars, including before and after egg production.

**Results:**

These results showed that expressed genes differed significantly between individuals grown under high and low food levels and among individuals of different instars and genotypes. Among these differentially expressed genes (DEGs), we found 16 that changed expression level before resting egg production. Some of these genes showed high-level expression only before resting egg production and one gene was an ortholog of *bubblegum* (*bgm*), which is reportedly up-regulated before diapause in bumblebees. According to gene ontology (GO) enrichment analysis, one GO term annotated as *long-chain fatty acid biosynthetic process* was enriched among these 16 genes. In addition, GO terms related to glycometabolism were enriched among down-regulated genes of individuals holding resting eggs, compared to those before resting egg production.

**Conclusions:**

We found candidate genes highly expressed only before resting egg production. Although functions of candidate genes found in this study have not been reported previously in *Daphnia*, catabolism of long-chain fatty acids and metabolism of glycerates are related to diapause in other organisms. Thus, it is highly probable that candidate genes identified in this study are related to the molecular mechanism regulating resting egg production in *Daphnia*.

**Supplementary Information:**

The online version contains supplementary material available at 10.1186/s12864-023-09369-3.

## Background

Diapause is an important trait of various plants and animals to overcome unsuitable environments. In aquatic animals, diapause occurs at various developmental stages, from eggs to adults, depending on the taxon [1, 2]. Planktonic crustaceans of the genus *Daphnia*, commonly found in freshwater environments, diapause as resting eggs when environmental conditions become unfavorable. Under favorable conditions, they produce subitaneous eggs parthenogenetically, which develop into embryos soon after release into the maternal brood chamber. However, in response to environmental stimuli such as short photoperiod [3, 4], high population density [5], low food quality or quantity [4, 6, 7], appearance of predators [8, 9] or competitors (Maruoka et al., submitted for publication), mature females produce resting eggs that generally require fertilization. At that time, their carapaces are thickened and they develop a special form called ephippia, that helps protect resting eggs against predators, pathogens, harmful chemicals and harsh environmental conditions, such as drying or freezing [10]. Then, resting eggs are deposited into the ephippial carapace after fertilization by mating with a male [11]. Thus, appearance of *Daphnia* with an ephippial carapace indicates that they are about to initiate resting egg production. After release, epphipia sink to the lake bottom where they remain until environmental conditions once again become favorable [12, 13] or else they float to the surface to hitchhike on waterfowl for dispersal [14].

As mentioned above, many studies have investigated when and why *Daphnia* species produce resting eggs. However, our understanding of molecular mechanisms for induction of resting egg production in *Daphnia* is still limited. Based on RNA-seq analysis, one study found that the chitin_bind_gene family was up-regulated in *Daphnia similoides* that developed ephippial carapaces [15]. However, expression of genes involved in induction of resting eggs may occur before development of the ephippial carapace. Another study found six highly expressed genes in *Daphnia pulex* that produce resting eggs [16]. However, the study examined gene expression using mixtures of adolescent instars, which have not yet developed ephippial carapaces, and adult instars, which have already formed them. Therefore, candidate genes found in that study may be related to formation of the ephippial carapace rather than to induction of resting eggs themselves. To identify genes functionally associated with induction of resting egg production, it is necessary to examine changes in expression level between adolescent and adult instars.

Panarctic *Daphnia pulex* [17, 18] is an endemic species in North America and has expanded its distribution, even into Japan [19]. Unlike most other *Daphnia* species, some genetic lines of this species, including lineages in Japan, are obligate parthenoforms [19, 20] and produce resting eggs without mating [19, 21]. Thus, this species enables a direct comparison of gene expression patterns between individuals producing subitaneous and resting eggs without effects of male production and crossing. In addition, the complete genome sequence of *D. pulex* has already been published [22]. Hence, panarctic *D. pulex* is an ideal species for investigating the molecular mechanism of resting egg production.

In this study, therefore, we performed RNA-seq analysis with panarctic *D. pulex* to identify candidate genes related to induction of resting egg production. For this purpose, we reared two genotypes of panarctic *D. pulex*, named JPN1 and JPN2. Individuals of the JPN1 genotype produce subitaneous eggs under both high and low food levels, while those of the JPN2 genotype produce subitaneous eggs under high food levels, but produce resting eggs under low food levels. By comparing gene expression patterns between the same instars of different genotypes and between different instars of the same genotypes grown under the same food levels, we examined genes that promote resting egg production.

## Results

### Egg production under different food availabilities

**Fig. 1:**
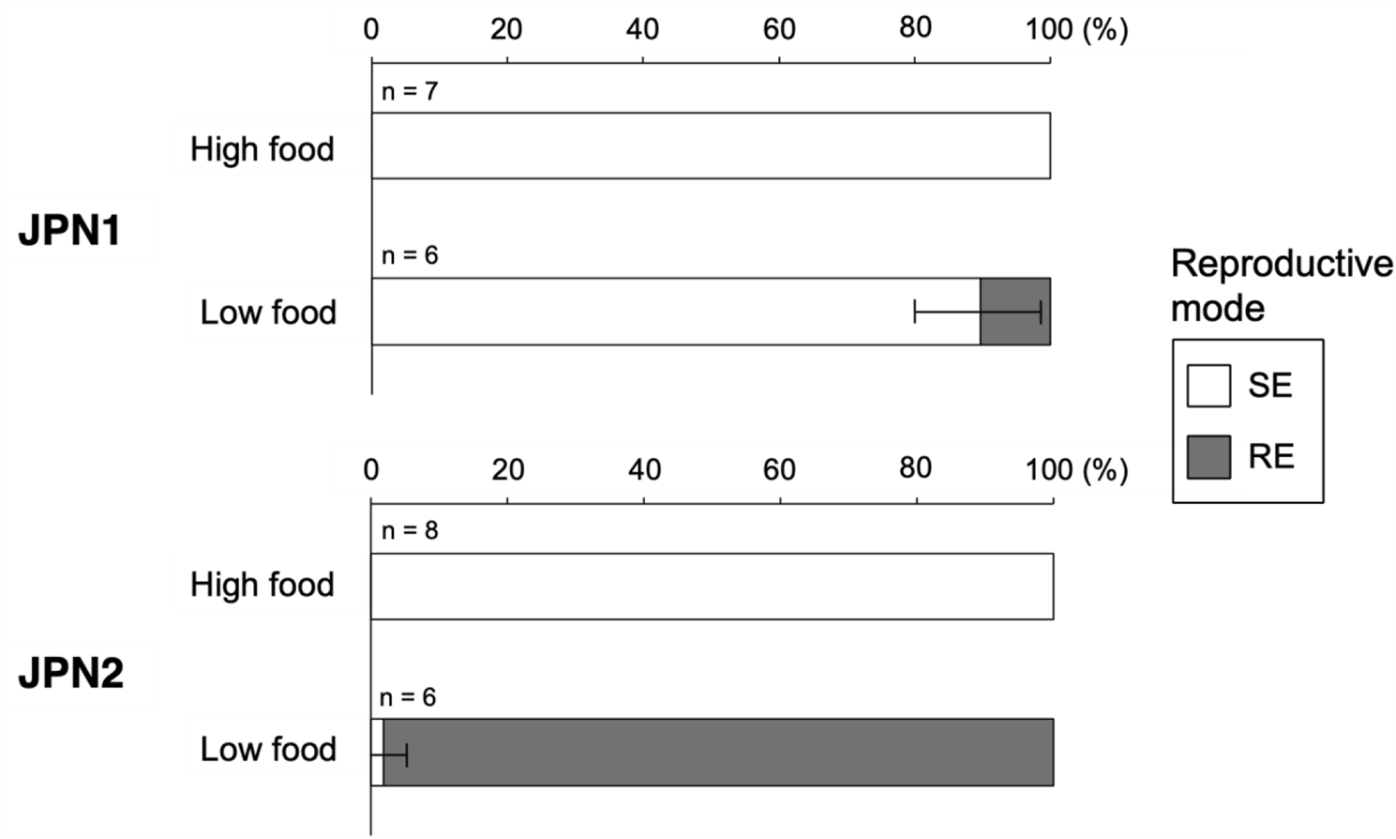
Frequency of reproductive modes at the first clutch (SE: subitaneous egg production, RE: resting egg production) of 10 individuals of panarctic *D. pulex* JPN1 and JPN2 genotypes, grown under high and low food levels. Error bars show the standard deviation

In both the JPN1 and JPN2 genotypes, all individuals produced subitaneous eggs when grown at a high food level of 2.0 mg C L^− 1^ (Fig. 1). In the JPN1 genotype, 90% of individuals also produced subitaneous eggs when grown under a low food level of 1.0 mg C L^− 1^, although the remaining individuals formed ephippial carapaces and produced resting eggs (Fig. 1). However, in the JPN2 genotype, 98% of individuals formed ephippial carapaces and produced resting eggs at low food availability although the remaining 2% of individuals produced subitaneous eggs.

### Gene expression pattern

**Fig. 2:**
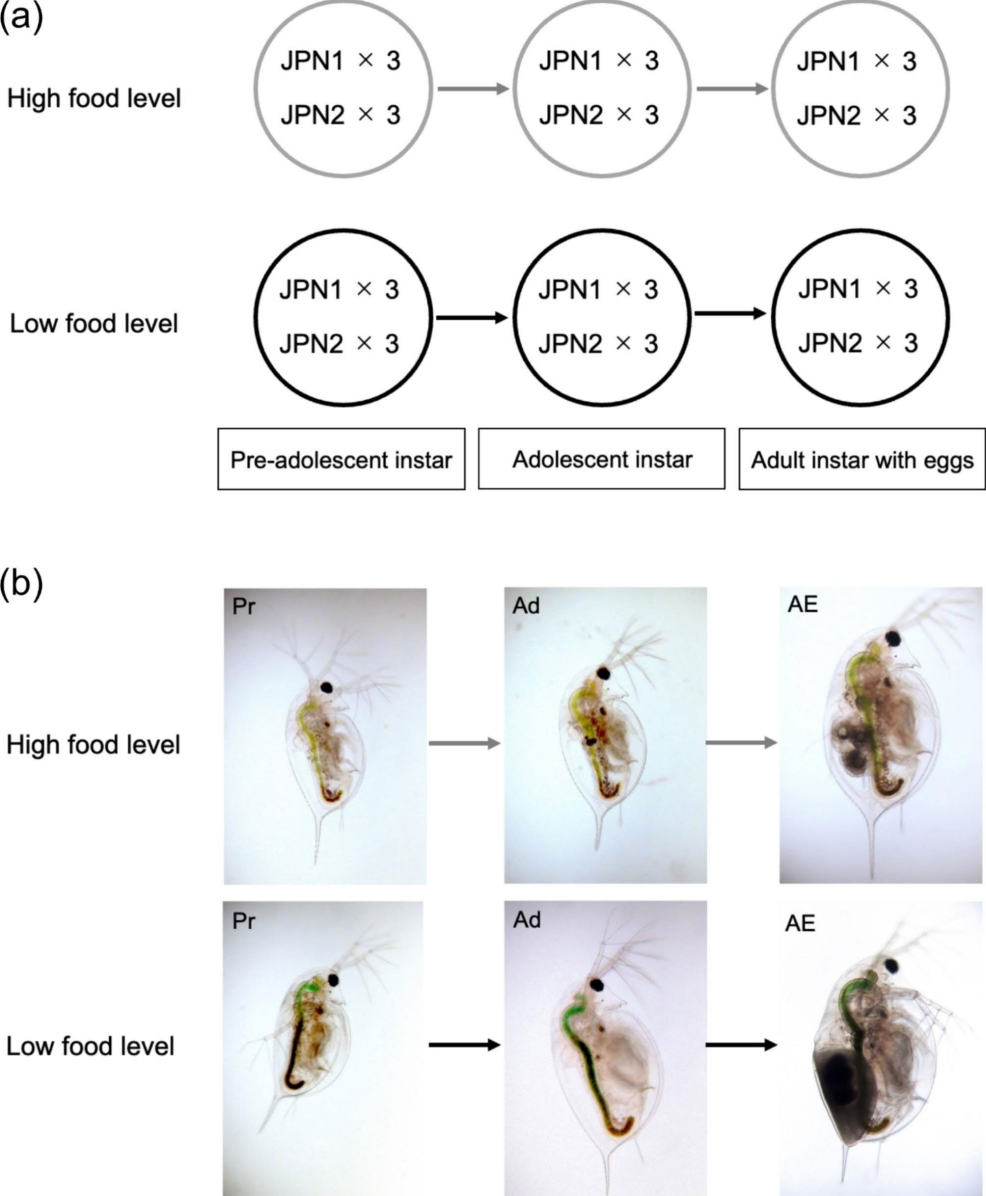
The experimental design of the RNA-seq analysis showing (**a**) the number of RNA sequences examined (2 food levels x 2 genotypes x 3 developmental instars x 3 replicate individuals) and (**b**) photographs of pre-adolescents, adolescents, and adults of the JPN2 genotype. Note that the ephippial carapace develops in adults (AE: JPN2 individuals) grown under the low food level

**Fig. 3:**
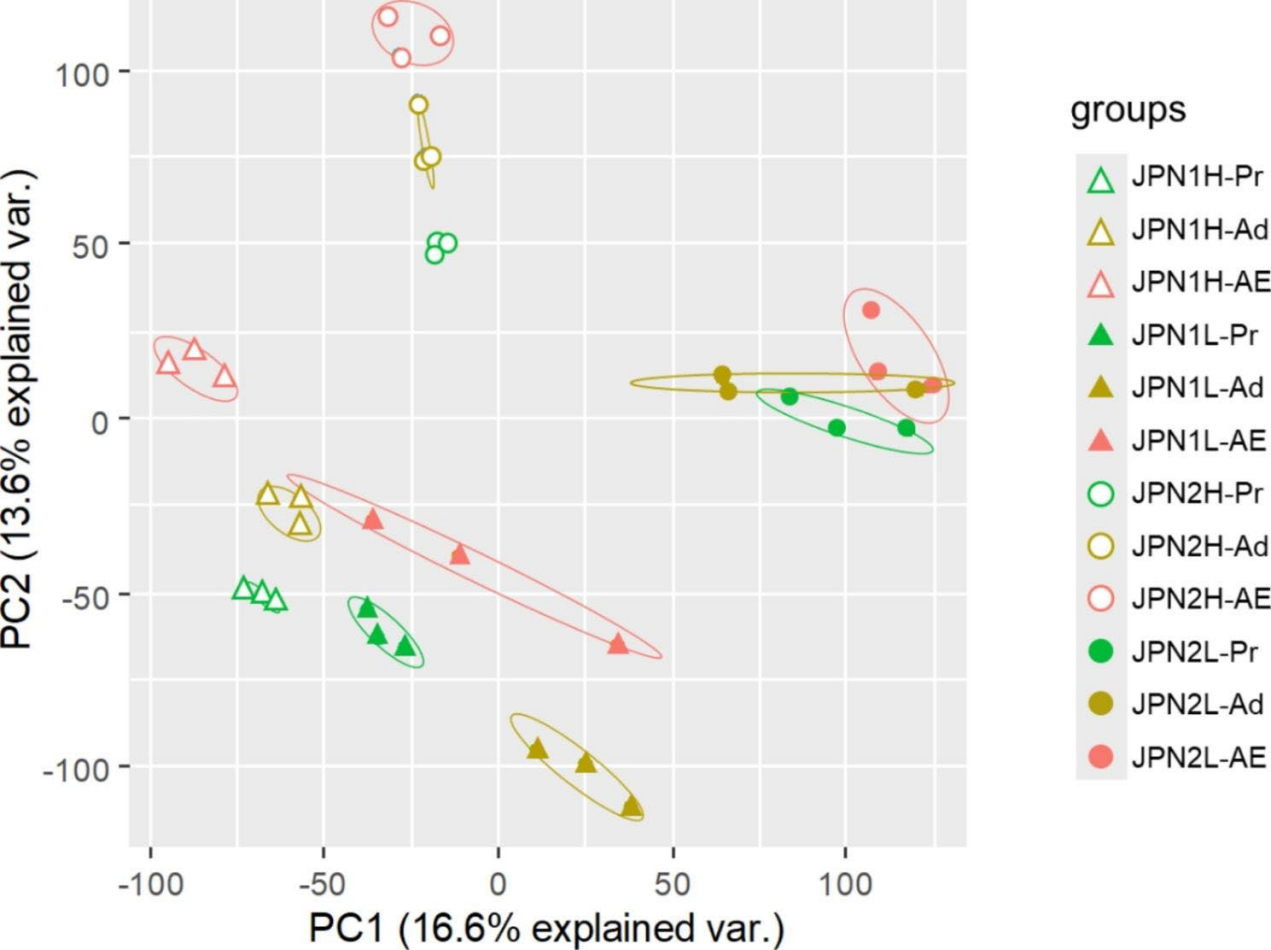
Results of principal component analysis (PCA) showing gene expression patterns among individuals sequenced. Expression patterns of the three different instars (Pr: pre-adolescent, Ad: adolescent and AE: adult with eggs) of the JPN1 and JPN2 genotypes grown under high (H) and low (L) food levels, denoted by different colors and symbols

For RNA sequencing, at both food levels, we collected JPN1 and JPN2 individuals of three developmental instars (Fig. 2). Pre-adolescent instars (Pr) that had not yet developed ovaries, adolescent instars (Ad) that had initiated ovary development, and adult instars (AE) that had deposited eggs into their brood chambers or ephippial carapaces. The dorsal part of the carapace becomes darker and develops into an ephippial carapace in adult instars, which produce resting eggs (Fig. 2). Using these individuals, 875,452,228 paired-end reads were obtained from 36 sequenced samples (2 genotypes x 3 developmental instars x 2 food levels x 3 replicate individuals). The percentage of reads that mapped to the reference genome ranged from 76 to 83% and was 79% on average. Principal component analysis (PCA) for relative expression levels, i.e., transcripts per million mapped reads (TPM), separated individuals into several groups (Fig. 3). In the JPN1 genotype, PC1 scores of gene expression patterns were similar among individuals under high and low food levels, regardless of instar. In the JPN2 genotype, however, PC1 scores differed between individuals at the two food levels. When fed abundant food, JPN2 showed similar PC1 scores to those of JPN1. However, PC1 scores differed between these genotypes under limited food. PC2 scores also differed among individuals of different genotypes and instars (Fig. 3).

### Genes differed in expression

**Fig. 4:**
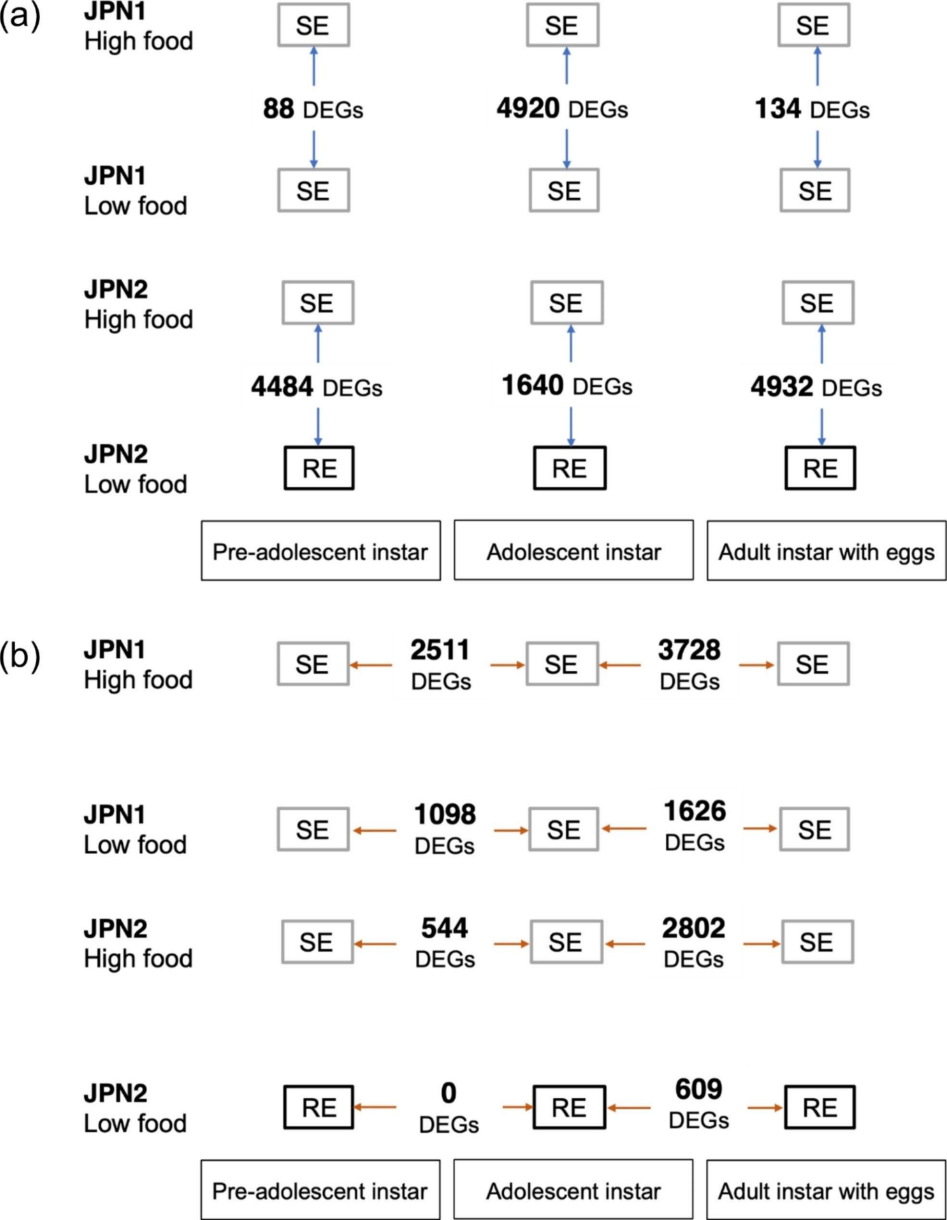
Differentially expressed genes (DEGs) obtained from RNA-seq analyses for two panarctic *D. pulex* genotypes grown under low and high food levels. Individuals producing subitaneous and resting eggs are denoted SE and RE, respectively. (**a**) Numbers of DEGs between individuals fed high and low food in each instar within genotypes. (**b**) Numbers of DEGs between different instars within genotypes grown under high and low food levels

To identify differentially expressed genes (DEGs) between individuals producing subitaneous and resting eggs, we first investigated DEGs between JPN2 genotypes of the same developmental instars fed high and low food diets (Fig. 4a). We found 4484, 1640, and 4932 significant DEGs between individuals under different food levels in pre-adolescent, adolescent, and adult instars, respectively (Fig. 4a). However, since the food level differed between those individuals, those DEGs should include genes related to growth and maintenance in response to changes in the food level, in addition to those related to differences in eggs produced. To remove growth- and maintenance-related genes, we examined DEGs between JPN1 genotypes fed high and low food diets and found 88, 4920, and 134 significant DEGs in pre-adolescent, adolescent, and adult instars, respectively (Fig. 4a). Genes common among DEGs in these JPN1 and JPN2 genotypes should be related to growth responses to changes in the food level. Therefore, we subtracted these common genes from DEGs in the JPN2 genotypes and obtained 4436, 479, and 4871 DEGs in pre-adolescent, adolescent, and adult instars, respectively.

Then, to identify genes related to induction of resting egg production, we further examined DEGs among the different JPN2 instars that produced resting eggs when food abundance was low. No significant DEGs differed between JPN2 pre-adolescent and adolescent instars fed low food, although we detected 544 DEGs that differed significantly from those of instars fed high food (Fig. 4b). We also investigated DEGs between JPN2 adolescent and adult instars and found 609 significant DEGs among those fed low food (Fig. 4b). These 609 DEGs should include genes related not only to resting egg production but also to growth and maintenance. To isolate genes unrelated to resting egg production, we examined DEGs between instars producing subitaneous eggs. We found 3728 and 1626 DEGs between JPN1 adolescent and adult instars fed high and low food, respectively, and 2802 DEGs between JPN2 adolescent and adult instars fed high food (Fig. 4b). Of these genes, the 231 genes were overlapped with the 609 DEGs identified from the difference between JPN2 adolescent and adult instars fed low food. Since these 231 DEGs were unlikely to be related to resting egg production, we removed them from the 609 DEGs. Then, we obtained the 378 DEGs that were specific to JPN2 fed low food.

As mentioned above, we found 479 unique DEGs that differed in expression level only in adolescent instar of the low food-fed JPN2 genotypes. These DEGs should include genes both related and unrelated to the resting egg production. The genes associated with the resting eggs are likely to have changed expression levels between adolescent and adult instars. As above, we found 378 unique DEGs that changed expression level between these instars of JPN2 fed low food. Thus, genes common to both sets, i.e., the 479 DEGs and the 378 DEGs, should be genes that changed expression level before resting egg production. There were only 16 such DEGs.

**Fig. 5:**
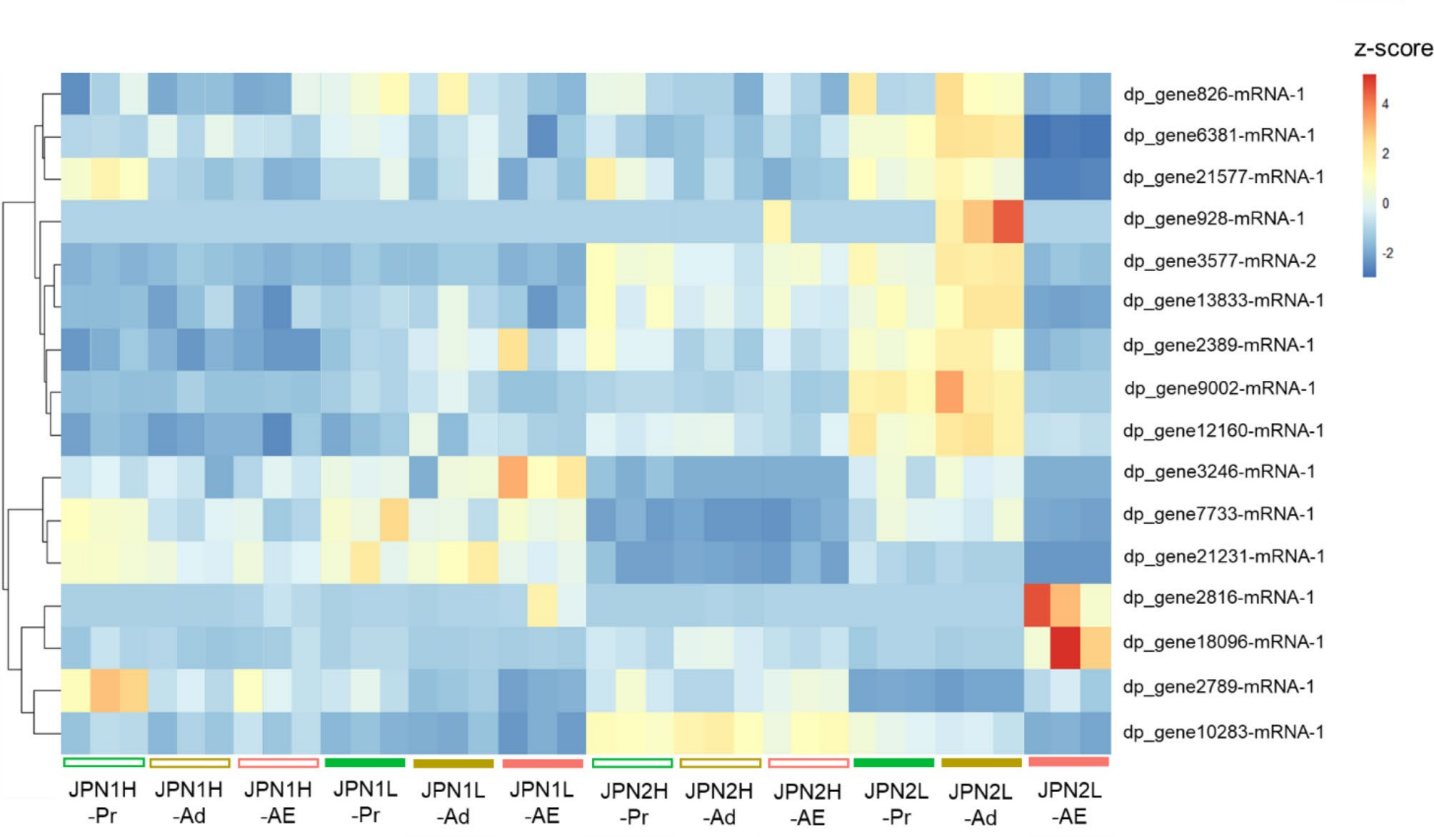
A heatmap showing the result of cluster analysis for relative expression levels of 16 DEGs that significantly changed expression levels before resting egg production. The three instars (Pr: pre-adolescent, Ad: adolescent and AE: adult with eggs) grown under high (H) and low (L) food levels are denoted by different colors on the horizontal axis. Note that individuals labeled JPN2L produced resting eggs with high frequency. In the heatmap, more up-regulated genes and more down-regulated genes are denoted by brighter red and brighter blue colors, respectively

For these 16 DEGs, we performed cluster analysis to determine which genes were similar in overall expression among the 36 sequenced samples. The results are illustrated with a heat map and a dendrogram (Fig. 5) and box plots (Fig S1), which showed that relative expression levels of dp_gene826-mRNA-1, dp_gene928-mRNA-1, dp_gene3577-mRNA-2, and dp_gene13833-mRNA-1 were high only in JPN2 adolescent instars fed low food, which produced resting eggs. In particular, the gene dp_gene928-mRNA-1 was highly expressed in JPN2 adolescent instars fed low food and showed a mean TPM of 14.34. Relative expression levels of dp_gene6381-mRNA-1, dp_gene21577-mRNA-1, dp_gene2389-mRNA-1, dp_gene9002-mRNA-1, and dp_gene12160-mRNA-1 were high in both JPN2 pre-adolescent and adolescent instars fed low food. However, among these, the genes dp_gene21577-mRNA-1 and dp_gene2389-mRNA-1 were also highly expressed in some of JPN2 individuals fed high food and JPN1individuals (Fig. S1).

### GO enrichment analysis

To examine functional enrichment of the 16 DEGs, we first investigated gene ontology (GO) terms belonging to *biological process* (BP) and *molecular function* (MF) domains (Table S1). Then, according to results of the GO enrichment analysis, we found one significantly enriched GO term, *long-chain fatty acid biosynthetic process* (BP, GO:0042759) (*p* = 0.04). Genes annotated with this GO term were dp_gene6381-mRNA-1 and dp_gene928-mRNA-1 (Table S1). dp_gene6381-mRNA-1 showed a relatively high expression level in JPN2 pre-adolescent and adolescent instars fed low food (Fig. 5, Fig. S1), which produced resting eggs (Fig. 1). dp_gene928-mRNA-1 was relatively highly expressed in JPN2 adolescent instars fed low food (Fig. 5, Fig. S1). They produced resting eggs (Fig. 1).

We also investigated significantly enriched GO terms for the 479 DEGs containing genes that exhibited different expression levels between JPN2 adolescent instars producing subitaneous eggs and those producing resting eggs. Of these, 226 DEGs were up-regulated and 253 were down-regulated in individuals producing resting eggs compared to those producing subitaneous eggs (Fig. S2). Unlike the analysis above, we found no significant enriched GO term in either DEG group.

We also performed GO enrichment analysis for the 378 DEGs that exhibited different expression levels between adolescent and adult instars. Of these, 239 DEGs were up-regulated and 139 were down-regulated in adult instars compared to adolescent instars (Fig. S3). Among the up-regulated genes, no significantly enriched GO terms were found. However, among down-regulated genes, seven GO terms were significantly enriched (Table 1). These included *hydrolase activity* (MF, GO:0016787) contained *hydrolase activity, hydrolyzing O-glycosyl compounds* (MF, GO:0004553), *hydrolase activity, acting on glycosyl bonds* (MF, GO:0016798), *carboxylic ester hydrolase activity* (MF, GO:0052689) and *glucosidase activity* (MF, GO:0015926).

**Table 1:** Enriched GO terms among down-regulated genes that changed expression levels significantly between JPN2 adolescent and adult instars grown under low food

Down-regulated genes at the adult instar					
domain	name	ID	ratio in study	ratio in pop	*p*
BP	carbohydrate metabolic process	GO:0005975	15/139	514/26,018	0.00028
MF	hydrolase activity	**GO:0016787**	41/139	3056/26,018	1.17E-05
MF	hydrolase activity, hydrolyzing O-glycosyl compounds	**GO:0004553**	13/139	232/26,018	9.88E-07
MF	hydrolase activity, acting on glycosyl bonds	**GO:0016798**	13/139	250/26,018	1.23E-06
MF	carboxylic ester hydrolase activity	**GO:0052689**	8/139	175/26,018	0.00242
MF	glucosidase activity	**GO:0015926**	4/139	30/26,018	0.00697
MF	extracellular matrix structural constituent	GO:0005201	7/139	165/26,018	0.00976

BP: biological process, MF: molecular function

Bold ID of GO terms were annotated in the same gene

## Discussion

To identify genes related to resting egg production in *Daphnia*, we compared gene expression patterns of two genotypes that differ in reproductive modes, even under the same food conditions. In addition, since it was likely that resting egg production was initiated before formation of ephippial carapaces and appearance of ovaries, we compared gene expression patterns of pre-adolescent, adolescent and adult instars with these genotypes. Through these comparisons, we found 16 differentially expressed genes (DEGs) that changed expression levels between before and after resting egg production.

Among the 16 DEGs, dp_gene6381-mRNA-1 and dp_gene928-mRNA-1 were highly expressed only before resting egg production. Especially, TPM values of dp_gene928-mRNA-1 were zero for most individuals except before resting egg production (adolescent instars). In genomic BLAST, we could not find the description of these genes and thus their function because of the paucity of studies investigating the function of genes in *Daphnia* [23]. However, we found that *ceramidase* (*CDase*) and *bubblegum* (*bgm*) are orthologs to dp_gene6381-mRNA-1 and dp_gene928-mRNA-1, respectively, among genes in *Drosophila melanogaster*, a model insect. Ceramidase, the enzyme encoded by *CDase* cleaves fatty acids from ceramide to produce sphingosine [24]. Ceramide and sphingosine function as signaling molecules that regulate cell differentiation, growth, and apoptosis [25–27]. The other gene, *bgm*, functions in the biosynthesis of long-chain fatty acids [28, 29]. A previous study found an interaction between lipid metabolism and sleep [30]. In fact, Thimgan et al. [31] reported that mutant *bgm* alters sensitivity to sleep deprivation in *D. melanogaster*. In bumblebees (*Bombus terrestris*), an ortholog to *bgm* was up-regulated before diapause and was down-regulated during diapause [32], which is consistent with the present results. Recently, GPR120, a new family of G protein-coupled receptors (GPCRs), was identified as a plasma membrane receptor that uses fatty acids as its natural ligand [33]. Since then, fatty acids have been recognized not only as nutritional materials, but also as signaling molecules [34]. Thus, the biosynthesis of fatty acids may be important as signaling molecules for initiating diapause in *Daphnia*.

We also examined gene description and orthologs of the other DEGs specific to the adolescent instars of JPN2 using genomic BLAST, but could not find information that was useful to estimate the function of these genes. Instead, we found that the 16 DEGs, including dp_gene6381-mRNA-1 and dp_gene928-mRNA-1, were significantly enriched in *long-chain fatty acid biosynthetic process* (BP, GO:0042759) in GO enrichment analysis. Fatty acids, especially highly unsaturated fatty acids, are known to be important nutritional substances for the growth and reproduction in zooplankton [35, 36]. In addition, a study showed that resting eggs of *Daphnia* contain higher levels of polyunsaturated fatty acids, such as linolenic acid and eicosapentaenoic acid, than subitaneous eggs [37]. These results suggest that long-chain fatty acids are necessary to produce resting eggs and to maintain their long-term viability [37].

In this study, we found no DEGs between pre-adolescent and adolescent instars of the JPN2 genotype fed a low food and producing resting eggs. According to a cluster analysis of the 16 genes, the gene expression pattern of JPN2 genotypes was similar between these two developmental instars on low food. These results suggest that resting egg production was promoted continually in these two instars. Note that in the present experiment, we collected individuals of each instar according to the number of molts. Thus, one may wonder if the lack of significant DEGs was due to a mixture of individuals that may have differed in their physiological conditions. However, this possibility would not explain why a large number of DEGs were observed between these instars in the case of the JPN1 genotype.

Unlike the comparison of pre-adolescent and adolescent instars, we found 378 unique DEGs between adolescent and adult instars of the JPN2 genotype fed low food. Since the JPN2 genotype produced resting eggs on low food, these genes are likely associated with resting egg production. Of these, 239 genes were up-regulated in adult instars relative to the adolescent instars. These up-regulated genes included *Dpep*-*1, 2*, and *3*, which were reported as up-regulated genes in *D. pulex* producing resting eggs relative to producing subitaneous eggs [16]. According to findings of the foregoing study, *Dpep-1-3* are expressed in epidermal cells of the carapace where the ephippium is formed [16]. Thus, the 239 up-regulated genes mentioned above may be related to development of the ephippial carapace.

Among the 378 DEGs found between JPN2 adolescent and adult instars fed low food, 139 genes were down-regulated in adult instars relative to adolescents. According to GO enrichment analysis, these 139 down-regulated genes were enriched in seven GO terms (Table 1). Among these GO terms, *hydrolase activity* (MF, GO:0016787), *hydrolase activity, hydrolyzing O-glycosyl compounds* (MF, GO:0004553) and *hydrolase activity, acting on glycosyl bonds* (MF, GO:0016798) is upper-hierarchy of *glucosidase activity* (MF, GO:0015926). These GO terms pertain to the glucosidase that releases glucose. Thus, the 139 down-regulated genes are thought to relate to carbohydrate metabolism. Alpha-glucosidase is involved in metabolizing glycogen for energy production in animal tissues [38] and beta-glucosidase is involved in digestion of cellulose in *Daphnia* [39]. Down-regulation of genes annotated as having glucosidase activity in adult instars implies reduction of glucosidase activities when eggs are deposited into ephippia. In other words, glucosidase activities increase before resting egg production. Thus, individuals producing resting eggs may have increased glucosidase activity to produce energy for resting eggs under limited food. However, there is another possible explanation for increased glycometabolism. In silkworms (*Bombyx mori*), which also diapause at the egg stage, conversion of glycogen to sorbitol and glycerol was reported in resting eggs [40, 41]. Glycerol helps to protect insects from winter cold [42]. Although the conversion of glycogen into glycerol has not yet been reported in *Daphnia*, the present study suggests that the change in expression levels of genes related to glycometabolism is involved in resting egg production of *Daphnia*.

Several studies have shown that genes related to the circadian clock, such as *period*, *timeless* or *cycle*, are related to diapause in invertebrates such as *D. melanogaster* [43], the parasitic wasp, *Nasonia vitripennis* [44], the butterfly, *Pararge aegeria* [45] and *Daphnia magna* [46]. In addition, a rhodopsin photoreceptor is responsible for regulating the frequency of resting egg production in *D. magna* [47]. In the present study, however, we did not find genes related to the circadian clock or rhodopsin photoreceptor among the genes whose expression revels change specifically resting eggs production. Induction of resting egg production was not affected by day length in genotypes of panarctic *D. pulex* used in the present study (Maruoka, personal observation), and these genes were not detected in relation resting egg production.

## Conclusions

The present study found some candidate genes and gene groups related to induction of resting egg production in panarctic *D. pulex*. Our results suggest that biosynthetic process of long-chain fatty acid and glycometabolism are related to resting egg production. However, it remains unclear whether changes in expression of these genes control resting egg production. In future studies, functions of these genes should be examined at a phenotypic level by manipulating expression levels using molecular techniques.

## Methods

### Genotypes of panarctic *Daphnia pulex*

We used JPN1 and JPN2 genotypes of panarctic *D. pulex* that were originally collected at Lake Hataya Ohnuma (38°14’43.6"N, 140°12’16.4"E). These genotypes have been maintained for more than five years in a temperature-controlled room at 20 °C with COMBO medium [48] and *Scenedesmus obliquus* (algae) as food. To prepare specimens for experiments, each genotype was individually grown at 20 °C with COMBO medium in a 50-mL glass bottle with 2.0 mg C L^− 1^ of algal food.

### Induction of resting egg production

For both the JPN1 and JPN2 genotypes, 10 neonates from the 2nd or 3rd clutches of females in the pre-culture above were randomly collected and grown in 300 mL of COMBO medium with 2.0 mg C L^− 1^ of algal food. Resting egg production occurs when individuals were placed at a food level lower than that experienced by their mothers [6]. Therefore, to induce resting egg production, we transferred 10 neonates from pre-culture to a small bottle with 50 mL of COMBO medium and 1.0 mg C L^− 1^ as a low food level. For comparison, we transferred 10 neonates from pre-cultures to a bottle with 300 mL of COMBO medium with 2.0 mg C L^− 1^ as a high food level. In both conditions, the light intensity was set to 10 to 20 µE m^− 2^ S ^− 1^ for full days. Media and food in the bottles were replaced every other day for the first several days, but when the *Daphnia* matured into adults, we added algal food every day.

### Animal collection for RNA sequencing

According to our observation, panarctic *D. pulex* molt approximately once every two to three days at 20 °C and this species deposited subitaneous eggs in the brood chamber after the fourth molt (the 5th instar) under high food levels. As in other *Daphnia* species [49–52], ovaries do not appear during the pre-adolescent instar (the 3rd instar), but appear and develop to produce subitaneous or resting eggs during next adolescent instar (the 4th instar). Then, they deposit mature eggs when they molt to the adult instar (the 5th instar). However, the number of molts can vary depending on genotypes and food levels. In this study, under high food, specimens of JPN1 and JPN2 genotypes deposited subitaneous eggs at the 5th instar and 6th instar, respectively. Under low food, animals of the JPN1 genotype deposited subitaneous eggs and those of the JPN2 genotype deposited resting eggs at the 7th instar. Therefore, at both low and high food levels, we collected specimens of all three developmental instars, pre-adolescent (Pr), adolescent (Ad) and adult instar (AE), depending on the instar at which they deposited eggs. Notably, genes related to molting change during a molt cycle in *Daphnia*. For example, chitobiase, an enzyme involved in recovery of chitin from the old exoskeleton increases from 42 to 60 h after molting [53]. In addition, the ecdysteroid level (molting hormone) increases almost 30 h after molting [54]. Therefore, we collected individuals within 24 h after molting, avoiding the immediate post-molt period, when expression levels of genes involved in molting are likely to be less variable. For each developmental instar, we pooled 10 individuals per sample for RNA extraction and assayed samples in triplicate. Thus, we prepared 36 sequenced samples, consisting of three developmental instars (Pr, Ad and AE), two genotypes (JPN1 and JPN2), and two food levels (high and low), each with three replicates (Fig. 2).

### RNA extraction and sequencing

*Daphnia* were stored in RNAlater® at − 28 °C until RNA extraction. Before RNA extraction, 10 individuals per rearing condition were transferred to 1-Thioglycerol / Homogenization Solution and were ground with a motor-assisted pestle (Biomasher II, NIP, Japan) until particulate debris was no longer observed. RNA was extracted using a Maxwell RSC Plant RNA Kit with a Maxwell® RSC Instrument AS4500 (Promega, Madison, WI, USA) following the manufacturer’s protocol and RNA concentrations were measured with a NanoDrop (Thermo Fisher Scientific Inc., MA, USA). RNA quality was checked with an Agilent 2100 Bioanalyzer using an Agilent RNA 6000 Nano Kit (Agilent Technologies, Inc., Santa Clara, CA, USA). RNA concentrations of the final solutions ranged from 24 to 177 ng µL^− 1^. For RNA sequencing, more than 1.0 µg of RNA was prepared. RNA solutions were stored at − 80 °C until sequencing.

RNA sequencing and library construction were performed by Novogen (Pledran, France) under mediation by Filgen, Inc. (Aichi, Japan). Sequencing of 150-bp paired-end reads was performed on an Illumina NovaSeq6000 sequencing system (Illumina, Inc., San Diego, CA, USA).

### Transcriptome analysis

Quality control of raw Illumina RNA-seq paired-end reads was performed using “fastp” version 0.20.1 [55]. If more than 40% of the bases in at least one of the reads were low-quality (< QV30), we removed both reads. In addition, adapter sequences were discarded. We mapped read data to the *D*. *pulex* genome assembly PA42.4.1 available at GenBank under accession GCA_900092285.2 [56, 57] with “HiSat2” version 2.2.1 [58]. Then we converted bam files using “Samtools” version 1.9 [59], and performed transcript assembly with the annotation file in the supporting information File S1 in [57]. Finally, we estimated the gene expression level as TPM (transcripts per million mapped reads), normalized by transcript length, of each transcription product using “Stringtie” version 2.1.5 [60].

To obtain a visual overview of differences in gene expression among genotypes with different instars and food levels, principal component analysis (PCA) was performed on calculated TPM values using the package “tidyverse” [61] and “fs” [62] of R version 4.1.0. The result of PCA was then plotted with the package “ggbiplot” [63], in R version 4.1.0.

To investigate differentially expressed genes (DEGs) between individuals grown under high and low food levels and among individuals of different instars, read count data were normalized using the Trimmed Mean of M-values (TMM) normalization method included in “edgeR” [64] with the package “TCC” [65] of R, version 4.1.0. TMM normalization was conducted to adjust skewed expression of genes that were unique to, or highly expressed in one experimental condition [66]. Then, with the normalized data set, differential expression analysis was conducted with “edgeR” [64] and the package “tidyverse” (R, version 4.1.0). In this analysis, to define significant DEGs, a false discovery rate (FDR) of 0.05 was used as the significance level.

To analyze similarity of expression patterns in DEGs identified as having significantly changed expression levels, we performed a cluster analysis for TPM after standardization with a mean of zero and a standard deviation of 1 using the package “tidyverse” (R, version 4.1.0). Then, the result was visualized with a heatmap, drawn using the package “pheatmap” [67] (R, version 4.1.0).

To clarify functional enrichment of DEGs, we used Gene ontology (GO) terms [68, 69] annotated to genes of *D. pulex* V1.0 [22] in the Ensembl dataset. Among domains of GO terms, we used GO terms categorized into *molecular function* (MF) and *biological process* (BP) for GO analysis. Annotation of transcripts of the *D. pulex* genome assembly PA42.4.1 [56, 57], was performed with an Ensembl BLASTp search (https://metazoa.ensembl.org/Multi/Tools/Blast) against *D. pulex* V1.0 [22]. In addition, to determine GO term frequency across DEGs, GO enrichment analysis was performed using Python GOATOOL [70] with a corrected *p*-value < 0.05 as a threshold to determine significance. We also used GOATOOL [70] to examine hierarchical structure among these enriched GO terms.

## Electronic supplementary material

Below is the link to the electronic supplementary material.

Supplementary Material 1

## Data Availability

Raw sequence data analyzed during the current study are available in the DDBJ Sequenced Read Archive under the accession numbers of DRA015695.
