# Supplementary material for: RNA-seq analysis to identify genes related to resting egg production of panarctic Daphnia pulex
Source: BMC Genomics. 2023 May 17;24:262. doi: 10.1186/s12864-023-09369-3 (PMC10190107; doi:10.1186/s12864-023-09369-3)
Supplement: Supplementary file 1 — Supplementary Material 1 [file 12864_2023_9369_MOESM1_ESM.pdf]

Supplementary Materials

RNA-seq analysis to identify genes related to the resting egg production of panarctic *Daphnia pulex*

Natsumi Maruoka[1,2], Takashi Makino[1], Jotaro Urabe[1]

1. Graduate School of Life sciences, Tohoku University, 6-3 Aoba, Aramaki, Aoba-ku, Sendai, Miyagi 980-8578, Japan, 022-795-6686

2. Present address: Center for Bioscience Research and Education, Utsunomiya University, 350 Mine-machi, Utsunomiya, Tochigi 321-8505, Japan, 028-649-5129

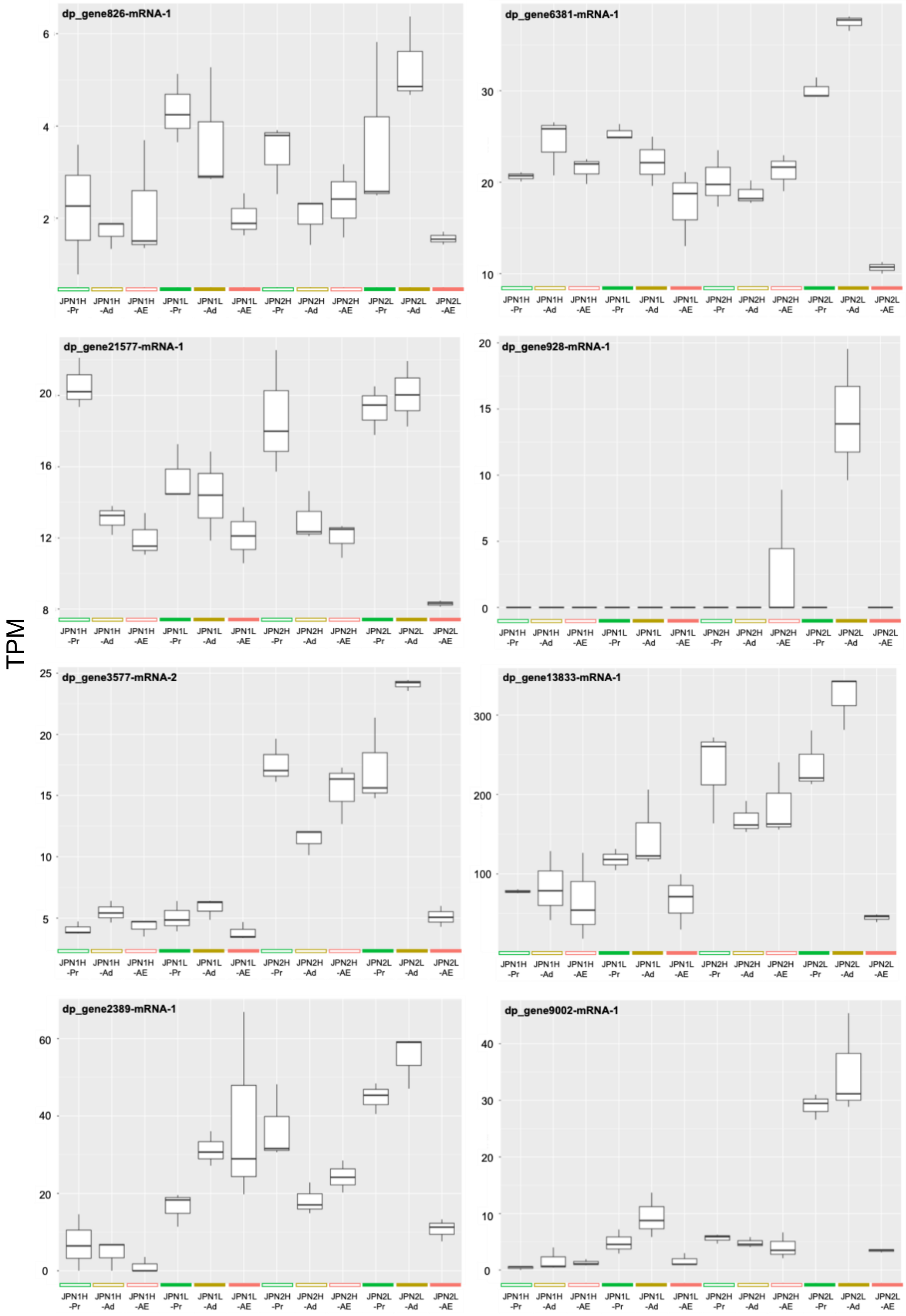

Figure S1. continued.

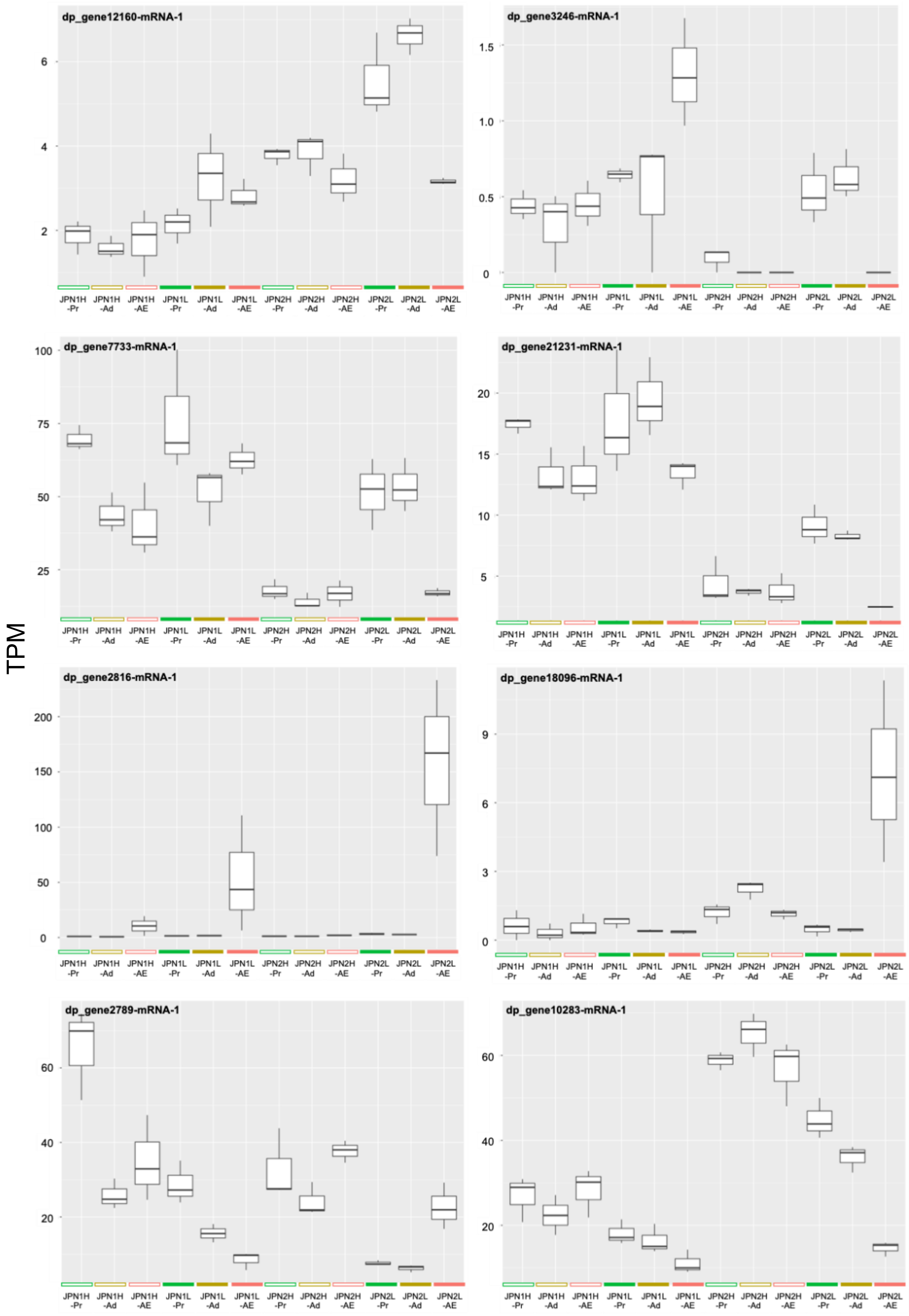

Figure S1. Box plots showing median, 25 and 75% quantiles, and range of the TPM values for 16 genes in 12 different individuals: preadolescent (Pr), adolescent (Ad) and adult individuals (AE) of JPN1 and JPN2 genotypes fed low (L) and high food (H). Among these individuals, only JPN2 individuals fed low food produced the resting eggs.

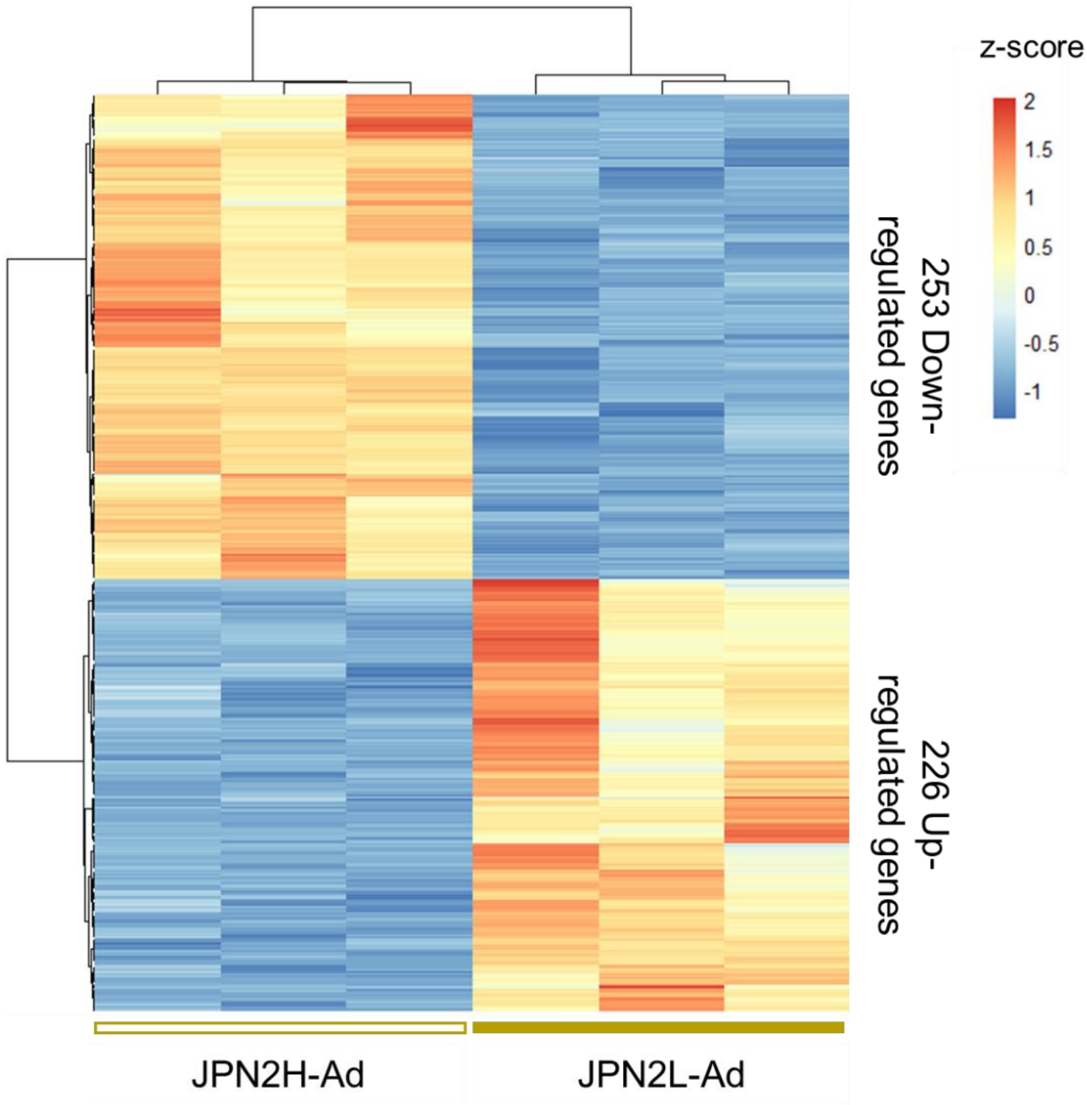

Figure S2. A heatmap showing the result of cluster analysis for relative expression level of 479 DEGs that differed the expression level between the individuals of adolescent (Ad) instar producing the subitaneous (H: high food level) and resting eggs (L: low food level). In the heatmap, bright red shows up-regulated genes and bright blue shows down-regulated genes.

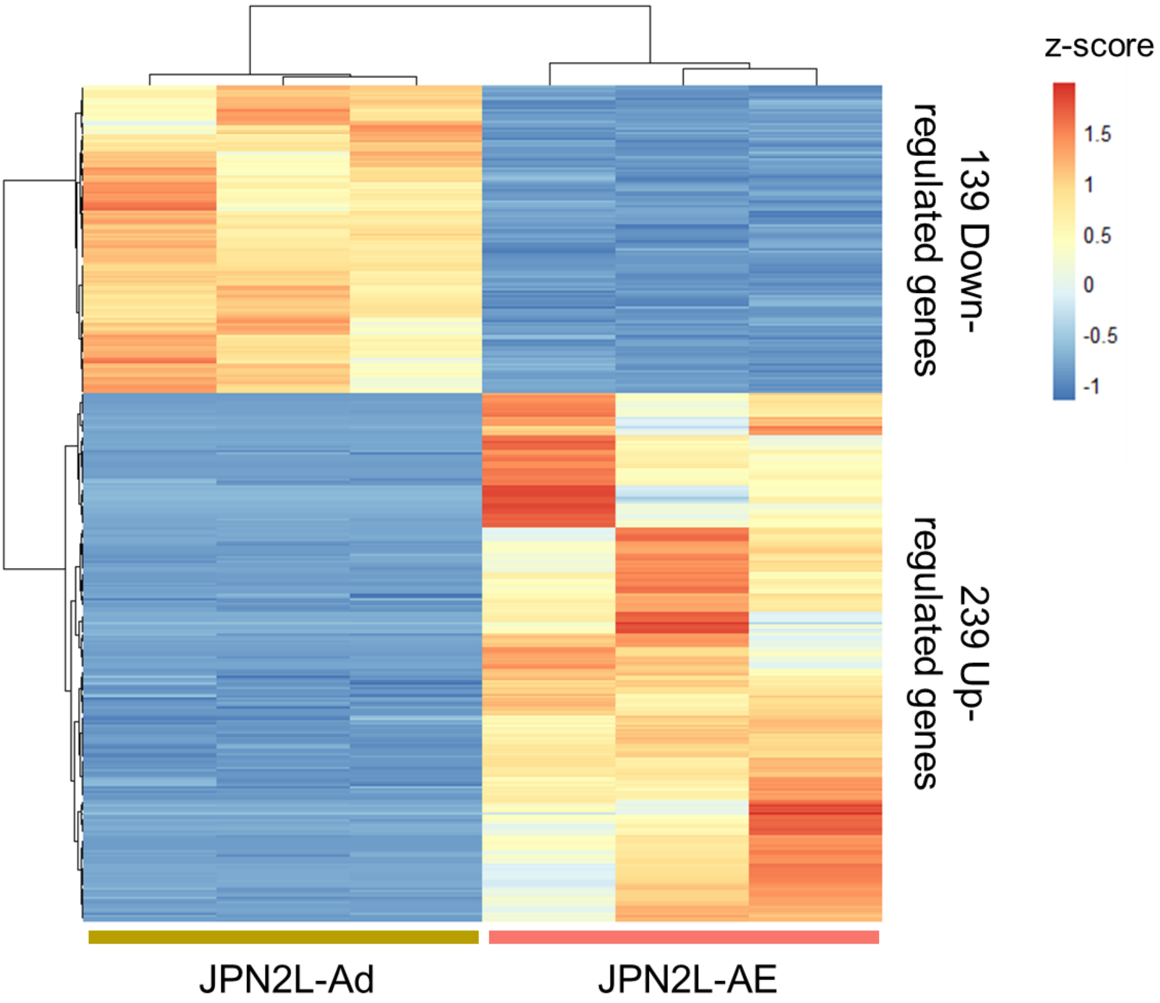

Figure S3. A heatmap showing the result of cluster analysis for relative expression levels of 378 DEGs that changed the expression levels between adolescent (Ad) and adult (AE) instar of JPN2 individuals producing resting eggs (L: low food level). In the map, more up-regulated genes and more down-regulated genes are denoted by brighter red and brighter blue colors, respectively.

Table S1. GO terms of the 16 genes that changed expression levels between before and after resting egg production.

Gene name of PA42.1.0	Ensembl BLAST	GO term accession	GO term name	GO domain
dp_gene826-mRNA-1	DAPPUDRAFT_313963	n/a		
dp_gene6381-mRNA-1	DAPPUDRAFT_309109	GO:0016787	hydrolase activity	molecular_function
		GO:0017040	N-acylsphingosine amidohydrolase activity	molecular_function
		GO:0017040	N-acylsphingosine amidohydrolase activity	molecular_function
		GO:0102121	ceramidase activity	molecular_function
		GO:0006629	lipid metabolic process	biological_process
		GO:0042759	long-chain fatty acid biosynthetic process	biological_process
		GO:0046512	sphingosine biosynthetic process	biological_process
		GO:0046514	ceramide catabolic process	biological_process
		GO:0006665	sphingolipid metabolic process	biological_process
		GO:0046514	ceramide catabolic process	biological_process
dp_gene21577-mRNA-1	DAPPUDRAFT_200654	GO:1902936	phosphatidylinositol bisphosphate binding	molecular_function
dp_gene928-mRNA-1	DAPPUDRAFT_187637	GO:0003824	catalytic activity	molecular_function
		GO:0004467	long-chain fatty acid-CoA ligase activity	molecular_function
		GO:0016405	CoA-ligase activity	molecular_function
		GO:0008152	metabolic process	biological_process
		GO:0042759	long-chain fatty acid biosynthetic process	biological_process
dp_gene3577-mRNA-2	DAPPUDRAFT_101374	GO:0005201	extracellular matrix structural constituent	molecular_function
		GO:0005515	protein binding	molecular_function
dp_gene13833-mRNA-1	DAPPUDRAFT_227643	GO:0004190	aspartic-type endopeptidase activity	molecular_function
		GO:0004190	aspartic-type endopeptidase activity	molecular_function
		GO:0016787	hydrolase activity	molecular_function
		GO:0008233	peptidase activity	molecular_function
		GO:0006508	proteolysis	biological_process
		GO:0006508	proteolysis	biological_process
		GO:0030163	protein catabolic process	biological_process
		GO:0008219	cell death	biological_process
dp_gene2389-mRNA-1	DAPPUDRAFT_196131	GO:0008270	zinc ion binding	molecular_function
		GO:0070006	metalloaminopeptidase activity	molecular_function
		GO:0008237	metallopeptidase activity	molecular_function
		GO:0042277	peptide binding	molecular_function
		GO:0046872	metal ion binding	molecular_function
		GO:0016787	hydrolase activity	molecular_function
		GO:0008233	peptidase activity	molecular_function
		GO:0004177	aminopeptidase activity	molecular_function
		GO:0006508	proteolysis	biological_process
		GO:0006508	proteolysis	biological_process
		GO:0043171	peptide catabolic process	biological_process
dp_gene9002-mRNA-1	DAPPUDRAFT_114783	GO:0005201	extracellular matrix structural constituent	molecular_function
		GO:0005515	protein binding	molecular_function
dp_gene12160-mRNA-1	DAPPUDRAFT_224775	GO:0005201	extracellular matrix structural constituent	molecular_function
		GO:0005515	protein binding	molecular_function
dp_gene3246-mRNA-1	DAPPUDRAFT_244756	GO:0005515	protein binding	molecular_function
dp_gene7733-mRNA-1	DAPPUDRAFT_316837	n/a		
dp_gene21231-mRNA-1	DAPPUDRAFT_309221	n/a		
dp_gene2816-mRNA-1	DAPPUDRAFT_315904	GO:0016491	oxidoreductase activity	molecular_function
		GO:0055114	oxidation-reduction process	biological_process
dp_gene18096-mRNA-1	DAPPUDRAFT_262661	GO:0016758	transferase activity, transferring hexosyl groups	molecular_function
		GO:0006688	glycosphingolipid biosynthetic process	biological_process
dp_gene2789-mRNA-1	DAPPUDRAFT_315829	n/a		
dp_gene10283-mRNA-1	DAPPUDRAFT_112046	n/a		

The order of the genes is the same to that of the heatmap shown in Fig. 5.
